# Isolated transverse process fracture of the lumbar vertebrae

**DOI:** 10.4103/0974-2700.55350

**Published:** 2009

**Authors:** Amit Agrawal, Sandeep Srivastava, Anand Kakani

**Affiliations:** Department of Surgery, Datta Meghe Institute of Medical Sciences, Sawangi (Meghe), Wardha, India; 1 Department of Orthopedics, Datta Meghe Institute of Medical Sciences, Sawangi (Meghe), Wardha, India

**Figure 1:**
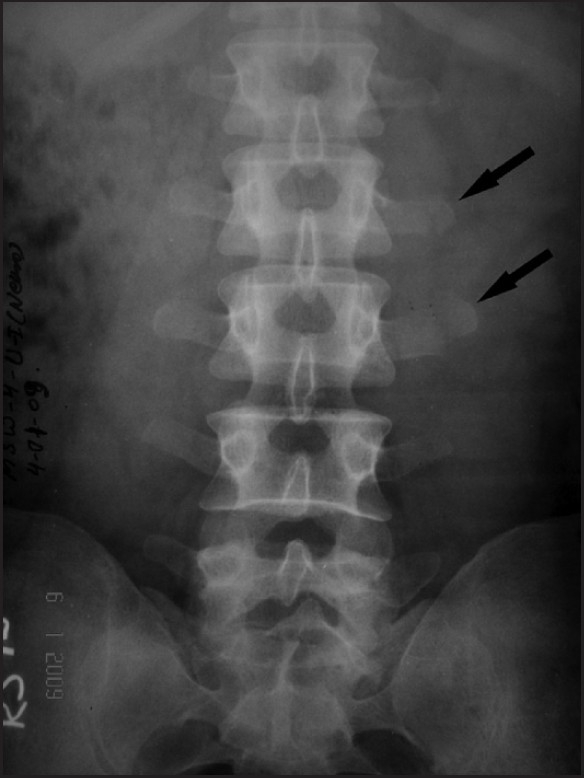
X-ray lumbar spine antero-posterior view showing the fractures of the left L2 and L3 transverse processes (arrows)

Sir,

It has been commonly thought that lumbar transverse process fractures as compared with body, pedicle and lamina fractures are minor injuries.[1] A 40-year gentlemen presented with a history of a fall while working from about 10 feet height. There was history of loss of consciousness for 15 min and low back pain. There was no history of vomiting, seizures, abdominal pain, hematuria or ear and nasal bleed. On examination, the patient was of average built. There was no pallor, icterus or cyanosis. Pulse was 73 beats/min, blood pressure was 118/70 mm/Hg and respiratory rate was 18 breaths/min. The heart sounds were normal and the air entry was equal in both the lungs. Abdomen was soft and non-tender. Bowel sound was normally heard. There was no hepatosplenomegaly. The patient was conscious, alert and oriented to time, place and person. Cranial nerves normal. Motor power was normal in all four limbs and there were no sensory deficits. Deep tendon reflexes were normally elicited and plantars were flexor. Chest and pelvic compression was negative. The patient could pass urine normally. There was local pain over the back on left side with mild scoliosis convexity to the left side. His X-ray of the lumbar spine showed the fractures of the tips of the transverse processes of the L-2 and 3 vertebrae on left side [Figure 1]. There were no other injuries. Ultrasonography of the abdomen was normal. The patient was kept nil by mouth for 24 h and supplemented with intravenous fluids and analgesics. He was mobilized with the support of a lumbar belt. He recovered completely, discharged on the seventh day and was doing well at follow-up. The present case prompted us to review the relevant literature regarding the management of isolated fracture of the lumbar vertebral transverse process. In English literature, it has been well documented that the lumbar vertebral transverse process fractures usually occur after higher energy traumas and must be evaluated extensively as it may be associated with serious other visceral injuries.[1–3] Fractures of the transverse processes of the lumbar vertebrae may result from direct blunt trauma (as in motor vehicle accidents), violent lateral flexion-extension forces (as in sports particularly football), avulsion of the psoas muscle or Malgaigne fractures of the pelvis.[1,3,4] Fractures of the transverse processes of the lumbar vertebrae usually occur as the result of major forces and a statistically significant association between transverse process fractures and abdominal injury has been reported.[5] Although transverse fracture of the lumbar vertebra can be a marker of associated visceral injuries including trauma to the abdominal viscera, retroperitoneum, spine, long bones and cranium,[3,5] the fractures of the transverse processes of the lumbar spine can occur in the absence of other vertebral and visceral damage.[6] In literature, in patients with isolated transverse fractures, the detailed investigations are recommended to exclude these associated visceral injuries. Classically, the conventional lumbar radiographs are used for evaluation of patients who are at risk of injury from blunt trauma; however, it is relatively insensitive in detecting the fractures as well as associated visceral injuries.[3,7] This insensitivity of conventional radiographs, especially in the setting of acute trauma can be due to the presence of overlying bowel gas, hence the full assessment and extent of fractures and associated injuries is not possible.[1,5,7] With the widespread use of whole-body CT scanning in trauma patients, the isolated transverse process fractures has been increasingly recognized and apart form this, the CT scan can detect the associated injuries in detail.[3,5,8] As in the present case, the transverse processes of the lumbar spine, not associated with neurological deficits or structural instability, can be managed conservatively without neurosurgical or orthopedic intervention.[8] The detailed radiological investigations should be performed wherever facilities and resources permit.[3,8,9] In a large retrospective review at Level I trauma center, it was found that where no other vertebral fracture is seen on an adequate screening CT scan, investigation may reasonably end. Also further imaging and consultations can lead to prolonged log-roll precautions, which delay the mobilization and can be potentially deleterious to overall patient care.[9] This can be followed in a setting with limited resources (either the resources are not available, e.g. CT scan or inability to perform recommended investigation in all the cases, e.g. financial constraints and poor affordability) and clinically stable patients can be carefully observed over an extended period.
